# Zoonotic Transfer of Clostridium difficile Harboring Antimicrobial Resistance between Farm Animals and Humans

**DOI:** 10.1128/JCM.01384-17

**Published:** 2018-02-22

**Authors:** C. W. Knetsch, N. Kumar, S. C. Forster, T. R. Connor, H. P. Browne, C. Harmanus, I. M. Sanders, S. R. Harris, L. Turner, T. Morris, M. Perry, F. Miyajima, P. Roberts, M. Pirmohamed, J. G. Songer, J. S. Weese, A. Indra, J. Corver, M. Rupnik, B. W. Wren, T. V. Riley, E. J. Kuijper, T. D. Lawley

**Affiliations:** a Section Experimental Bacteriology, Department of Medical Microbiology, Leiden University Medical Center, Leiden, Netherlands; b Host-Microbiota Interactions Laboratory, Wellcome Trust Sanger Institute, Hinxton, United Kingdom; c Centre for Innate Immunity and Infectious Diseases, Hudson Institute of Medical Research, Clayton, Victoria, Australia; d Department of Molecular and Translational Sciences, Monash University, Clayton, Victoria, Australia; e Cardiff School of Biosciences, Sir Martin Evans Building, Cardiff, United Kingdom; f Pathogen Genomics, Wellcome Trust Sanger Institute, Hinxton, United Kingdom; g Public Health Wales, Microbiology, Wales, United Kingdom; h Institute of Translational Medicine, University of Liverpool, Liverpool, United Kingdom; i Department of Veterinary Science and Microbiology, The University of Arizona, Tucson, Arizona, USA; j Department of Pathobiology, Canada Veterinary College, University of Guelph, Guelph, Canada; k Institute of Medical Microbiology and Hygiene, Österreichische Agentur für Gesundheit und Ernährungssicherheit (AGES), Vienna, Austria; l Faculty of Medicine, University of Maribor, Maribor, Slovenia; m National Laboratory for Health, Environment and Food, Maribor, Slovenia; n Department of Pathogen Molecular Biology, London School of Hygiene and Tropical Medicine, University of London, London, United Kingdom; o Department of Microbiology, PathWest Laboratory Medicine, Queen Elizabeth II Medical Centre, Western Australia, Australia; p Microbiology & Immunology, School of Pathology & Laboratory Medicine, The University of Western Australia, Western Australia, Australia; University of Tennessee at Knoxville

**Keywords:** Clostridium difficile, RT078, intercontinental transmission, interhost transmission, accessory genome, One Health concept, antibiotic resistance

## Abstract

The emergence of Clostridium difficile as a significant human diarrheal pathogen is associated with the production of highly transmissible spores and the acquisition of antimicrobial resistance genes (ARGs) and virulence factors. Unlike the hospital-associated C. difficile RT027 lineage, the community-associated C. difficile RT078 lineage is isolated from both humans and farm animals; however, the geographical population structure and transmission networks remain unknown. Here, we applied whole-genome phylogenetic analysis of 248 C. difficile RT078 strains from 22 countries. Our results demonstrate limited geographical clustering for C. difficile RT078 and extensive coclustering of human and animal strains, thereby revealing a highly linked intercontinental transmission network between humans and animals. Comparative whole-genome analysis reveals indistinguishable accessory genomes between human and animal strains and a variety of antimicrobial resistance genes in the pangenome of C. difficile RT078. Thus, bidirectional spread of C. difficile RT078 between farm animals and humans may represent an unappreciated route disseminating antimicrobial resistance genes between humans and animals. These results highlight the importance of the “One Health” concept to monitor infectious disease emergence and the dissemination of antimicrobial resistance genes.

## INTRODUCTION

Over the past decade, Clostridium difficile has emerged as the primary cause of infectious antibiotic-associated diarrhea in hospitalized patients (1). Unlike other common health care-associated pathogens, C. difficile produces resistant spores that facilitate host-to-host transmission and enable long term survival and dispersal in the health care system and the wider environment (2). The emergence of epidemic C. difficile ribotype (RT) 027 (NAP1/ST-1), responsible for many large-scale hospital outbreaks worldwide (3, 4), has been linked to environmental spore contamination and the acquisition of fluoroquinolone resistance (5). Enhanced research focus on C. difficile in the aftermath of the C. difficile RT027 outbreaks has revealed other evolutionarily distinct C. difficile lineages, in particular C. difficile RT078 (NAP07-08/ST-11), that are now emerging for unknown reasons as significant human pathogens (6).

The “One Health” concept, which connects the health of humans to the health of animals and their shared environments, represents a relevant framework for understanding the emergence and spread of pathogens. C. difficile RT078 is commonly isolated from both humans and farm animals (7) and is increasingly recognized as a causative agent of both health care- and community-associated C. difficile infection (CDI) (8). This lineage typically affects a younger population (9) and results in higher mortality than infection by C. difficile RT027 (10). Standard genotyping tools have highlighted genetic similarities between human and animal C. difficile RT078 (11–13) strains, raising the possibility of zoonotic transmission (14). Nevertheless, the exact evolutionary and epidemiological relationships between human and animal C. difficile RT078 strains remain unknown due to the lack of discriminatory power of these typing methods and the clonal nature of C. difficile lineages. Recently, using whole-genome phylogeny, we reported that asymptomatic farmers and their pigs can be colonized with clonal C. difficile RT078 isolates, demonstrating evidence for spread between animals and humans (15).

## MATERIALS AND METHODS

### Collection of C. difficile strains.

C. difficile laboratories worldwide were asked to send a diverse representation of their C. difficile 078 collections to the Lawley Laboratory, hosted at the Wellcome Trust Sanger Institute. Sample shipping was coordinated by the Lawley Laboratory. After receiving all shipped samples, DNA extraction was performed batchwise by one person using the same protocol and reagents to minimize bias. Phenol-chloroform was the preferred method for extraction, since it provides high DNA yield and intact chromosomal DNA. The genomes of 182 strains designated C. difficile RT078 (/NAP07-08/ST-11) by PCR ribotyping (16) were sequenced and combined with our previous collection of 65 strains of C. difficile RT078 (15), making a total of 247 strains analyzed in this study. These 247 strains were collected between 1996 and 2012 and are comprised of representative strains from 4 continents (North America, Europe, Australia, and Asia). Of these strains, 183 were derived from humans, 59 from animals (pigs, cattle, horses, and poultry), 4 from foods and 1 from an environmental sample. Details of all sequenced strains are listed in Table S1 in the supplemental material, including the European Nucleotide Archive (ENA) sample accession numbers. Metadata of the C. difficile RT078 strains has been made freely publicly available through Microreact (https://microreact.org/project/rJs-SYgMe) (17).

### Bacterial culture and genomic DNA preparation.

C. difficile strains were cultured on blood agar plates (bioMérieux, The Netherlands) for 48 h, inoculated into liquid medium (brain heart infusion [BHI] broth supplemented with yeast extract and cysteine) and grown overnight (ca. 16 h) anaerobically at 37°C. Cells were pelleted and washed with phosphate-buffered saline (PBS), and genomic DNA preparation was performed using a phenol-chloroform extraction as previously described (18).

### DNA sequencing, assembly, and annotation.

Paired-end multiplex libraries were prepared and sequenced using an Illumina Hi-Seq platform with fragment size of 200 to 300 bp and a read length of 100 bp as previously described (19, 20). An inhouse pipeline developed at the Wellcome Trust Sanger Institute (https://github.com/sanger-pathogens/Bio-AutomatedAnnotation) was used for bacterial assembly and annotation. It consisted of *de novo* assembly for each sequenced genome using Velvet v. 1.2.10 (21), SSPACE v. 2.0 (22), and GapFiller v. 1.1 (23) followed by annotation using Prokka v. 1.5.1 (24).

### Construction and analysis of the pangenome.

We used the pangenome pipeline Roary (25) to identify the C. difficile RT078 pangenome. Roary takes annotated draft assemblies in GFF3 format that were produced by Prokka (24). Predicted coding regions were extracted from the input and converted to protein sequences. Partial sequences (>5% nucleotides unknown or sequence length less than 120 nucleotides) were filtered, and the remaining sequences were iteratively clustered with CD-HIT (Cluster Database at High Identity with Tolerance), beginning with a sequence identity of 100% and matching length of 100% and continuing down to a default sequence identity of 98%. One final clustering step was performed again with CD-HIT, with a sequence identity of 100% and leaving one representative sequence for each cluster in a protein FASTA file. This was followed by a comprehensive pairwise comparison with blastp on the reduced sequences, with a default sequence identity percentage of 95% and matching length of 100%. The pangenome embodies the core genome, defined as those genes present in at least 90% of the genomes, and the accessory genome, defined as those genes present in between 10% and 90% of the genomes. Rare variant genes, found in less than 10% of genomes, were discarded.

Core gene (*n* = 3,368) alignment, an output from Roary, was used to construct the phylogenetic structure of 248 C. difficile strains. Single nucleotide polymorphisms (SNPs) were extracted from the core gene alignment using SNP-sites (26). A maximum likelihood tree based on SNP alignment was constructed using FastTree with the settings -gamma and -gtr (27), and the tree was visualized with iTOL (28).

### Average nucleotide identity (ANI) analysis.

Using Roary analysis, C. difficile RT078 strains isolated from humans and animals with identical core genomes were extracted using an inhouse R script. ANI was calculated by performing pairwise comparison of genome assemblies of these C. difficile RT078 strains using MUMmer (29).

### Identification of antimicrobial resistance gene sequence.

Antimicrobial resistance genes were identified within the C. difficile RT078 genomes through comparison to the CARD database with the ARIBA (Antimicrobial Resistance Identification By Assembly) software (https://github.com/sanger-pathogens/ariba).

### Accession number(s).

The genomes sequenced in this study were deposited in the ENA under accession numbers ERS005819 to ERS005821, ERS005823, ERS005825 to ERS005827, ERS005829, ERS005830, ERS005834 to ERS005837, ERS005839, ERS005840, ERS005842, ERS005847, ERS138003 to ERS138016, ERS138019 to ERS138025, ERS138057, ERS138067, ERS138068, ERS138087 to ERS138090, ERS138092 to ERS138100, ERS138103 to ERS138105, ERS138107, ERS138115 to ERS138120, ERS138122, ERS138124 to ERS138127, ERS138130, ERS138132 to ERS138149, ERS138151, ERS138152, ERS138154, ERS1645161 to ERS1645168, ERS188478 to ERS188487, ERS188489, ERS188492 to ERS188495, ERS188498, ERS188502 to ERS188504, ERS188534, ERS188555 to ERS188567, ERS188569 to ERS188592, ERS188658 to ERS188667, ERS199743, ERS199744, ERS199746 to ERS199751, ERS199753, ERS199755, ERS199756, ERS199759, ERS199762, ERS199765, ERS199826, and ERS199827 (see Table S1 in the supplemental material).

## RESULTS AND DISCUSSION

**FIG 1:**
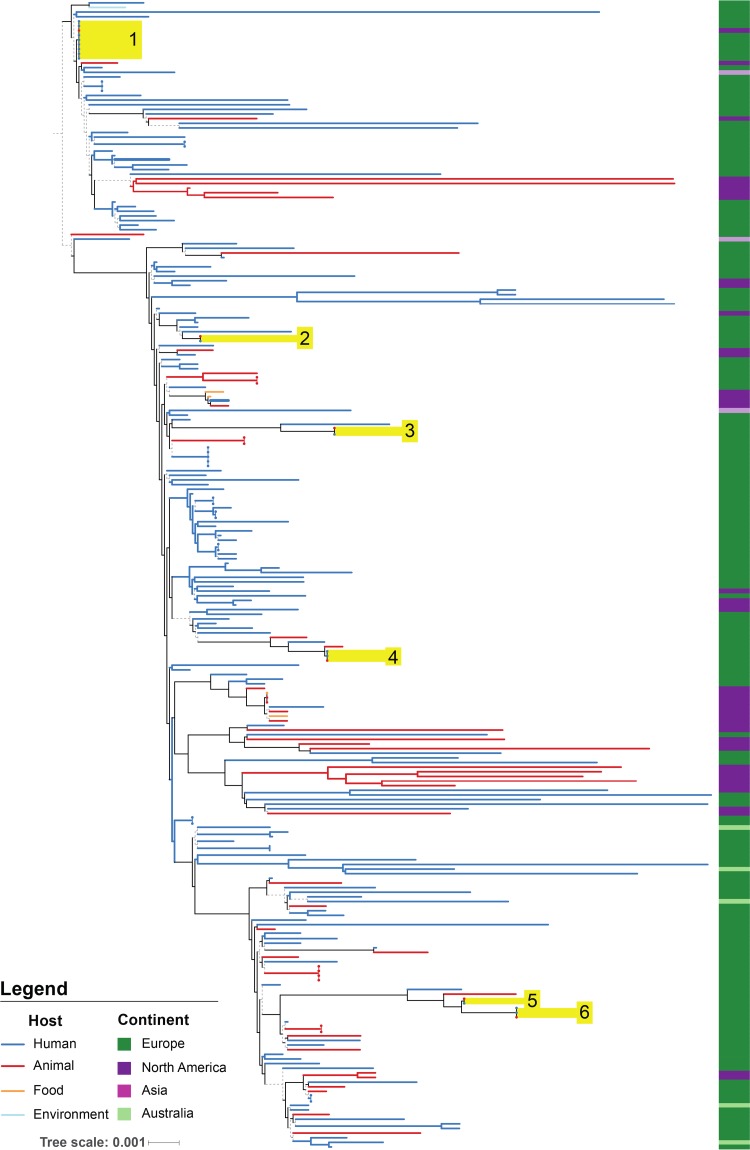
Phylogeography of human and animal Clostridium difficile RT078 strains. Maximum likelihood, midpoint-rooted phylogenetic tree of 248 genomes represents strains isolated from human (dark blue), animal (red), food (orange), and environmental (light blue) sources and collected from Europe (dark green), North America (purple), Asia (pink), and Australia (light green). Branches with bootstrap confidence values above 0.7 are shown as solid lines. The phylogeny demonstrates clear mixing of European and North American strains, indicating multiple transmission events between continents, and mixing of human and animal strains, indicating multiple transmissions events between these hosts. Closely related clusters (see Table 1) containing both human and animal isolates are labeled 1 through 6 and highlighted in yellow.

Here we assess the broad genetic diversity of C. difficile RT078, by performing whole-genome sequence analysis of 247 strains isolated predominantly from humans and animals that were collected from 22 countries across North America, Europe, Australia, and Asia between 1996 and 2012 (https://microreact.org/project/rJs-SYgMe) (Table S1). We explored the phylogenetic structure of C. difficile RT078 by generating a core genome maximum likelihood phylogeny that included the 247 C. difficile RT078 strains and the reference genome of C. difficile M120 (*n* = 248) (Fig. 1). Superimposing the geographic origin of strains revealed considerable coclustering of European (dark green) and North American (purple) strains across the phylogeny (Fig. 1). Permutation analysis on randomly generated equalized subsets of European (dark green) and North American (purple) genomes confirmed coclustering of geographically diverse strains (see Fig. S1 in the supplemental material). In addition, the absence of a single clade of C. difficile RT078 isolated in Australia (light green) is also suggestive of sporadic transmission between Europe and Australia (Fig. 1). Overall, the observed lack of geographic clustering is characteristic of repeated international transmission.

We next examined the phylogenetic distribution of strains isolated from humans (*n* = 184) and animals (*n* = 59) to understand the potential for zoonotic spread. This analysis identified examples of human to human and animal to animal spread and strong evidence of bidirectional spread of C. difficile RT078 between animals and humans across the phylogeny. These observations are supported by the extensive coclustering of human (blue lines) and animal strains (red lines) (Fig. 1). Focused analysis of closely related C. difficile RT078 strains identified 6 clusters containing both animal and human isolates with an identical core genome and highly similar whole genomes (ANI, ≥99.73%; Table 1). Surprisingly, Cluster 1 consists of an animal strain from Canada and human strains from the United Kingdom, indicating that zoonotic spread of C. difficile is not confined to a local population of humans and animals, as found previously (15). The existence of highly related human and animal isolates suggests that C. difficile RT078 has frequently spread between animals and humans.

**TABLE 1:** Six highly similar C. difficile RT078 clusters identified as identical through core genome analysis^*a*^

Cluster no.	ENA ID no.	Yr	Continent	Country	Host	ANI (%)
1	ERR171209	2004	North America	Canada	Animal	
	ERR171230	2010	Europe	United Kingdom	Human	99.93
	ERR256911	2011	Europe	United Kingdom	Human	99.91
	ERR171303	2008	Europe	United Kingdom	Human	99.90
	ERR256986	2012	Europe	United Kingdom	Human	99.84
	ERR256910	2011	Europe	United Kingdom	Human	99.83
	ERR1910469	1997	Europe	United Kingdom	Human	99.82
	ERR1910468	1997	Europe	United Kingdom	Human	99.80
	ERR256981	2008	Europe	United Kingdom	Human	99.75
2	ERR257071	2011	Europe	Netherlands	Animal	
	ERR257072	2011	Europe	Netherlands	Human	99.94
3	ERR257053	2011	Europe	Netherlands	Animal	
	ERR257057	2011	Europe	Netherlands	Human	99.77
4	ERR257067	2011	Europe	Netherlands	Animal	
	ERR171352	2011	Europe	Netherlands	Human	99.97
	ERR257052	2011	Europe	Netherlands	Human	99.91
5	ERR257046	2011	Europe	Netherlands	Animal	
	ERR257061	2011	Europe	Netherlands	Human	99.82
6	ERR257065	2011	Europe	Netherlands	Animal	
	ERR257044	2011	Europe	Netherlands	Human	99.80
	ERR257050	2011	Europe	Netherlands	Human	99.73

a All clusters contain isolates from both humans and animals. Average nucleotide identity (ANI) for human isolate compared to the animal isolate in the same cluster is also shown. ID, identification.

**FIG 2:**
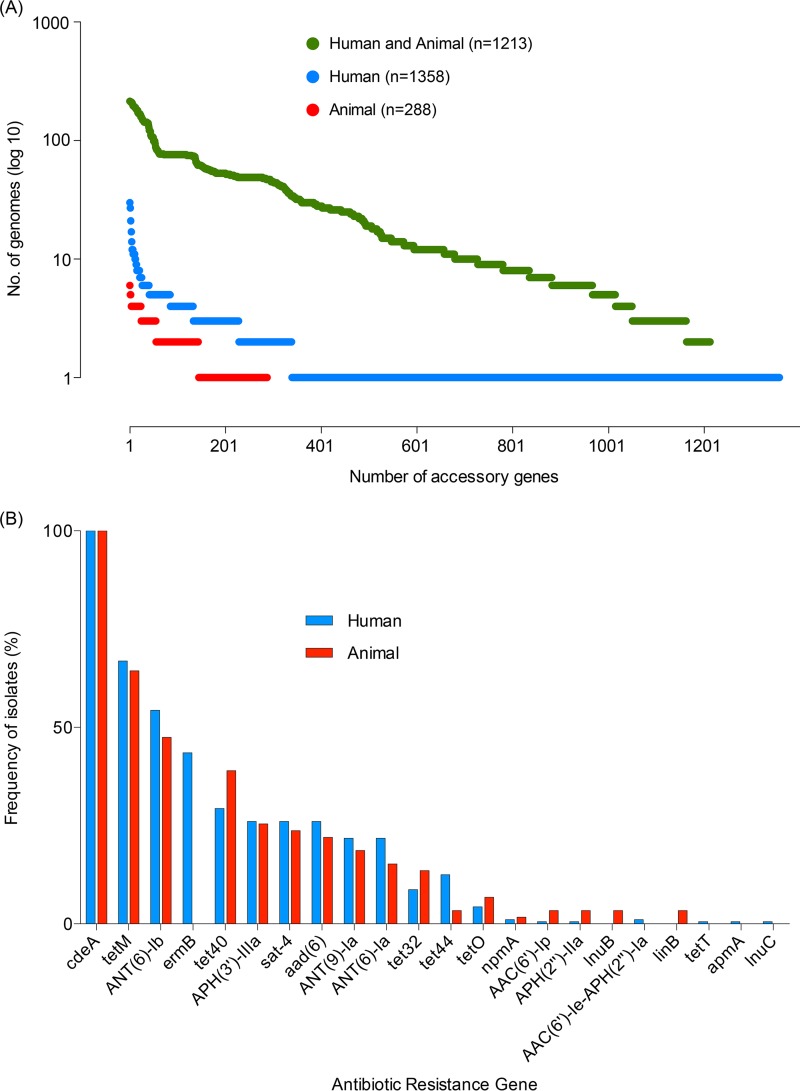
Indistinguishable accessory genome of C. difficile RT078 harbors a variety of antimicrobial resistance genes. (A) The accessory genes (*n* = 2,859) categorized according to host origin. The number of accessory genes (*x* axis) only found in human genomes (dark blue), only found in animal genomes (red), or found in both human and animal genomes (green) is plotted against the number of genomes in which these genes are present (*y* axis). (B) The frequency of predicted antimicrobial resistance genes (ARGs) within the 243 C. difficile RT078 strains. Human (dark blue) and animal (red) isolation sources are shown by color.

Next, a detailed analysis of the accessory genome, including mobile genetic elements, was performed to further explore the genomic similarities between human and animal strains. Of the 6,239 unique genes present across our genome collection, 3,368 genes (54.0%) were assigned to the core genome, leaving 2,871 genes (46.0%) present in the accessory genome (see Fig. S2 in the supplemental material). Considering only the human and animal isolates, 2,859 accessory genes were identified. The vast majority of human- and animal-specific accessory genes were found at low frequencies in the population (Fig. 2A). We observed no statistically significant difference in the number of strains carrying accessory genes exclusive to either the human or the animal population (χ^2^ test *P* value, 0.39). Considering only those accessory genes present in at least 10% of isolates (*n* = 465), 461 (99.1%) were identified in both human and animal isolates. The absence of accessory genes unique to either group either demonstrates that C. difficile has a stable accessory genome that is host-independent or provides further support for the frequent transmission of C. difficile between host populations.

Given the high percentage of mobile elements, including antimicrobial resistance genes harbored by C. difficile genomes (5, 6), we next sought to analyze distribution of different ARGs in the pangenome of human and animal strains. In total, 22 different putative ARGs are present in the 243 C. difficile RT078 genomes (Fig. 2B). The most common ARG was the chromosome-encoded *cdeA*, a well-known multidrug transporter that was detected in all strains; however, other common genes included those encoding resistance to aminoglycosides, tetracycline, and erythromycin (Fig. 2B). Importantly, no specific ARGs were statistically enriched in the animal isolates; however, the *ermB* (erythromycin resistance methylase) gene was identified in the human isolates (Fisher's exact test, *Q* value = 1.25 × 10^−07^). These results provide further support that a clonal C. difficile RT078 population containing a broad array of ARGs—exclusive of *ermB*, which has signs of unknown selective pressure in the human isolates—is spreading between humans and farm animals.

C. difficile is an ancient, genetically diverse species that has only emerged as a significant human pathogen over the past 4 decades. It remains to be determined why evolutionarily distinct lineages such as C. difficile RT027 and RT078 (6) are simultaneously emerging to cause disease in the human population. Previously, we have demonstrated that C. difficile RT027 acquired fluoroquinolone resistance during the 1990s in North America and rapidly spread through the global health care system (5). Here, we demonstrated that C. difficile RT078 has spread multiple times between continents, in particular between North America and Europe, highlighting that C. difficile emergence and spread is a global issue. In contrast to the distinct animal- and human-associated populations observed for the multidrug-resistant enteric pathogen Salmonella enterica serovar Typhimurium strain DT104 (30), we demonstrated that C. difficile RT078 is a clonal population moving frequently between livestock and human hosts, with no geographical barriers. Although the original reservoir remains unknown, the reciprocal transmission between humans and farm animals emphasizes the importance of a comprehensive One Health perspective in managing and controlling C. difficile RT078.

## Supplementary Material

Supplemental material
