# Supplementary material for: Zoonotic Transfer of Clostridium difficile Harboring Antimicrobial Resistance between Farm Animals and Humans
Source: J Clin Microbiol. 2018 Feb 22;56(3):e01384-17. doi: 10.1128/JCM.01384-17 (PMC5824051; doi:10.1128/JCM.01384-17)
Supplement: Supplemental material [file supp_56_3_e01384-17__index.html]

Supplemental material 

# Zoonotic Transfer of Clostridium difficile Harboring Antimicrobial Resistance between Farm Animals and Humans

## Supplemental material

- Supplemental file 1 -

  Fig. S1 (Permutation analysis of *Clostridium difficile* RT078 strains) and S2 (Pan-genome analysis) and Table S1 (*Clostridium difficile* RT078 isolates included in this study)

  PDF, 584K
