# Supplementary material for: Zoonotic Transfer of Clostridium difficile Harboring Antimicrobial Resistance between Farm Animals and Humans
Source: J Clin Microbiol. 2018 Feb 22;56(3):e01384-17. doi: 10.1128/JCM.01384-17 (PMC5824051; doi:10.1128/JCM.01384-17)
Supplement: Supplemental material [file JCM.01384-17_zjm999095844s1.pdf]

Supplementary information for:

Zoonotic transfer of *Clostridium difficile* harboring antimicrobial resistance between farm animals and humans.

C.W. Knetsch[1,†], N. Kumar[2,†], S.C. Forster[2,3,4], T.R. Connor[5], H.P. Browne[2], C. Harmanus[1], I.M. Sanders[1], S.R. Harris[6], L. Turner[7], T. Morris[7], M. Perry[7], F. Miyajima[8], P. Roberts[8], M. Pirmohamed[8], J.G. Songer[9], J.S. Weese[10], A. Indra[11], J. Corver[1], M. Rupnik[12,13], B.W. Wren[14], T.V. Riley[15,16], E.J. Kuijper[1] and T.D. Lawley[2,*]

*Correspondence to: Trevor Lawley, tl2@sanger.ac.uk

†These authors contributed equally to this work

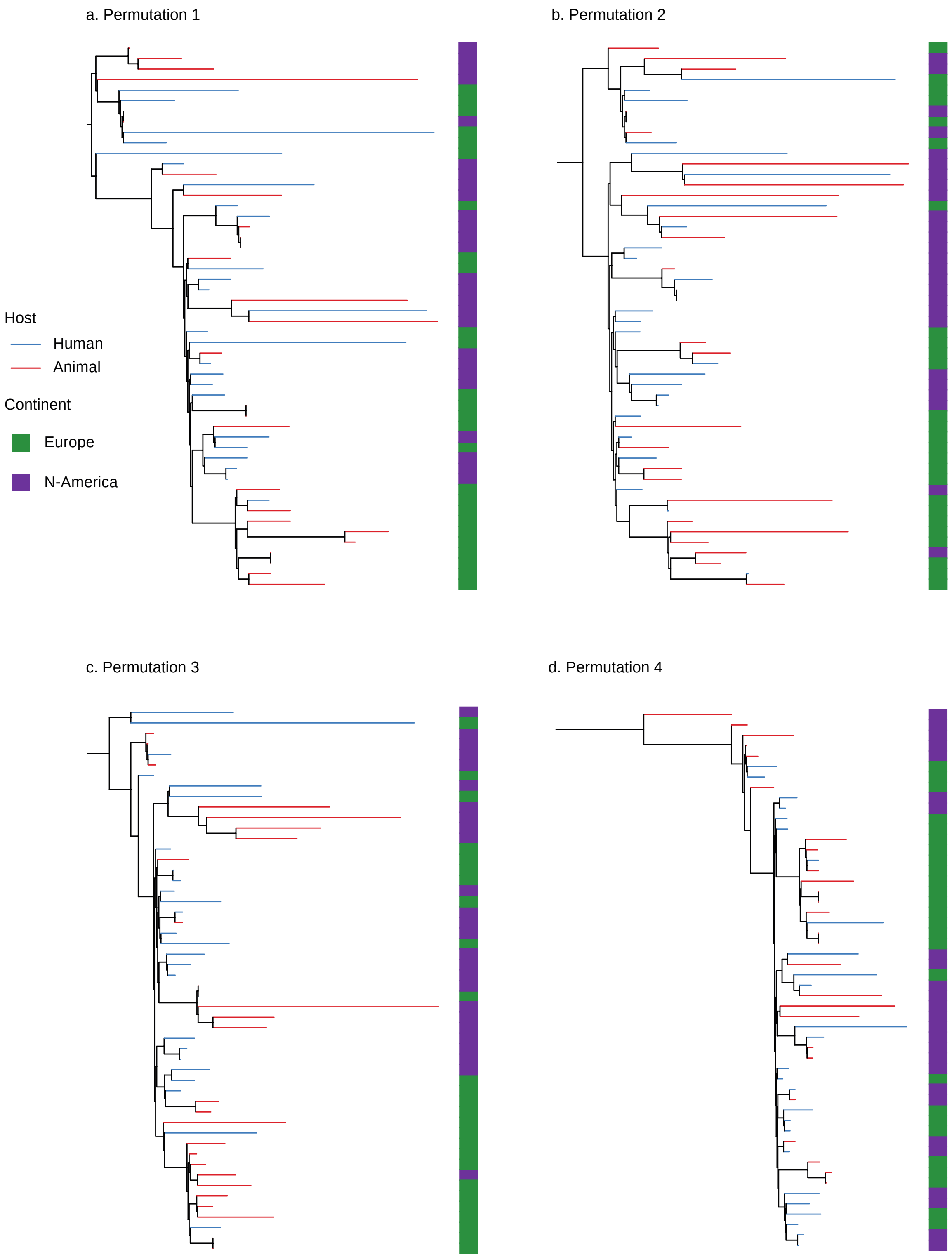
a. Permutation 1
b. Permutation 2
c. Permutation 3
d. Permutation 4
Host
Human
Animal
Continent
Europe
N-America

Figure S1. Permutation analysis of *C. difficile* RT078 strains. Four maximum likelihood phylogenies based on core genes alignment are shown. Each phylogeny includes randomly selected 52 *C. difficile* RT078 strains based on different hosts (human, 13 and animal, 13) and geographical locations (North America, 13 and Europe, 13). Strains isolated from food and environment and strains from Asia and Australia were not included due to limited number of strains. All four permuted phylogenies demonstrate similar patterns of mixing between human and animal strains as well as North American and European strains.

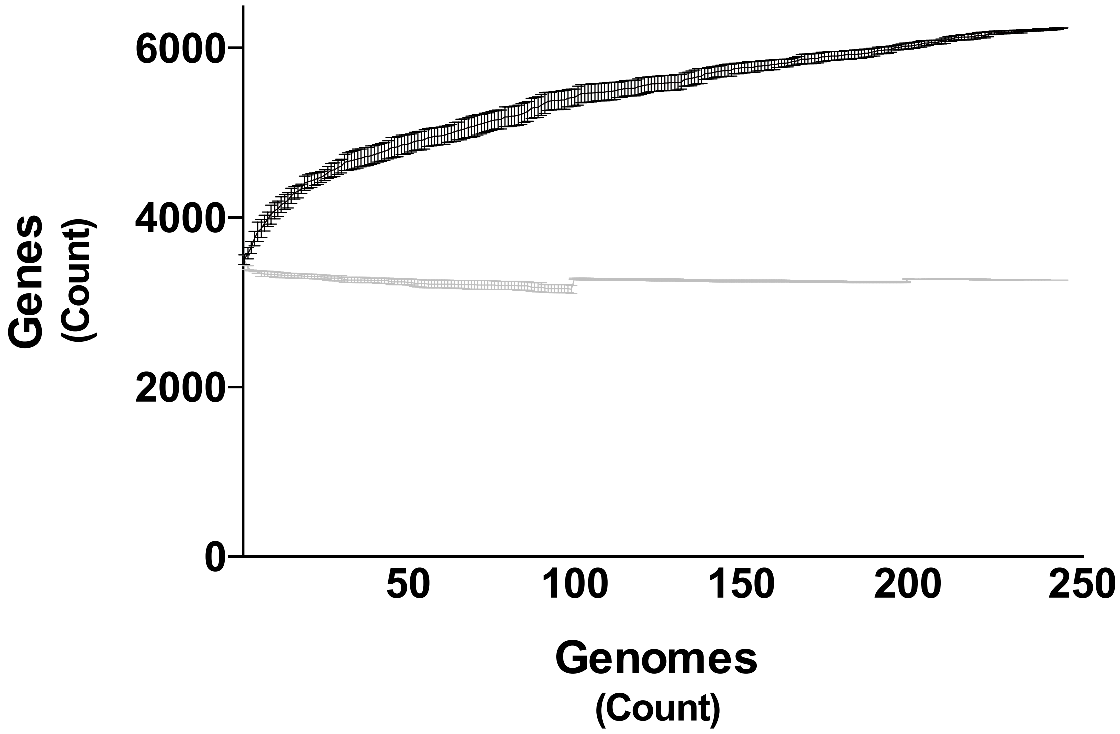

Figure S2. Pan genome analysis. Graph of pan genome gene content *C. difficile* RT078 strains increases (towards a total of 248 genomes; black line denotes the increasing pan genome, grey line denotes the core genome, the area between both lines is the accessory genome). The number of core genes (n=3,368) relative to the full complement of genes (pan genome; n=6,239; black) are indicated. Mean and 95% CI are plotted for randomly selected genome orders.

Table S1. *Clostridium difficile* RT078 isolates included in this study. ENA ids of genomes sequenced in this study are in blue and Knetsch et al; 2014 are in black.

ENA_ID	Continent	Country/site/study	City	Year	Source	Source_detail	Strain	Lab	Amount of sequence (MB)
ERS005819	Europe	UK	Belfast	2009	Human	Human	090692	Belfast	3.890666
ERS005820	Europe	UK	Belfast	2009	Human	Human	090374	Belfast	3.867971
ERS005821	Europe	UK	Belfast	2009	Human	Human	090323	Belfast	3.871589
ERS005823	Europe	UK	Belfast	2009	Human	Human	090099	Belfast	3.873315
ERS005825	Europe	UK	Belfast	2009	Human	Human	090571	Belfast	3.877374
ERS005826	Europe	UK	Belfast	2009	Human	Human	090574	Belfast	3.858835
ERS005827	Europe	UK	Belfast	2009	Human	Human	090608	Belfast	3.883906
ERS005829	Europe	UK	Belfast	2009	Human	Human	090372	Belfast	3.871356
ERS005830	Europe	UK	Belfast	2009	Human	Human	090668	Belfast	3.868649
ERS005834	Europe	UK	Belfast	2009	Human	Human	090652	Belfast	3.865859
ERS005835	Europe	UK	Belfast	2009	Human	Human	090482	Belfast	3.863124
ERS005836	Europe	UK	Belfast	2009	Human	Human	090600	Belfast	3.915472
ERS005837	Europe	UK	Belfast	2009	Human	Human	090021	Belfast	3.869025
ERS005839	Europe	UK	Belfast	2009	Human	Human	090445_B	Belfast	3.874636
ERS005840	Europe	UK	Belfast	2009	Human	Human	090445_A	Belfast	3.866392
ERS005842	Europe	UK	Belfast	2009	Human	Human	090468	Belfast	3.875479
ERS005847	Europe	UK	Belfast	2009	Human	Human	090205	Belfast	3.826134
ERS138003	Australia	Australia	Melbourne	2007	Human	Human	JIR_8535	Leiden-Kuijper	4.111489
ERS138004	Australia	Australia	Perth	2006	Human	Human	WA_94	Leiden-Kuijper	4.074605
ERS138005	Australia	Australia	Perth	2006	Human	Human	RPH_96	Leiden-Kuijper	3.959305
ERS138006	Australia	Australia	Melbourne	2007	Human	Human	JIR_8522	Leiden-Kuijper	3.994604

ERS138007	Europe	Austria	Feldkirch	2009	Human	Human	AT_1103	Leiden-Kuijper	3.982029
ERS138008	Europe	Belgium	Louvain	2009	Human	Human	BE_0104	Leiden-Kuijper	4.107547
ERS138009	Europe	Bulgaria	Sofia	2009	Human	Human	BG_1209	Leiden-Kuijper	3.962421
ERS138010	Europe	Denmark	Odense	2009	Human	Human	DK_1208	Leiden-Kuijper	4.005707
ERS138011	Europe	France	Rouen	2009	Human	Human	FR_0306	Leiden-Kuijper	3.999392
ERS138012	Europe	Germany	Freiburg	2009	Human	Human	DE_1210	Leiden-Kuijper	3.955792
ERS138013	Europe	Greece	Athens	2009	Human	Human	GR_0106	Leiden-Kuijper	3.96254
ERS138014	Europe	Hungary	Szeged	2009	Human	Human	HU_1104	Leiden-Kuijper	3.940605
ERS138015	Europe	Iceland	Reykjavik	2009	Human	Human	IS_0102-1	Leiden-Kuijper	3.88913
ERS138016	Europe	Ireland	Dublin	2009	Human	Human	IE_1101	Leiden-Kuijper	3.943131
ERS138019	Europe	UK	Leeds	2008	Human	Human	45/22	Leiden-Kuijper	3.897615
ERS138020	Europe	UK	Leeds	2008	Human	Human	R26231	Leiden-Kuijper	3.907054
ERS138021	Europe	Norway	Trondheim	2009	Human	Human	NO_1307	Leiden-Kuijper	3.958264
ERS138022	Europe	Portugal	Fonseca	2009	Human	Human	PT_1203	Leiden-Kuijper	3.992567
ERS138023	Europe	Slovenia	Ljubljana	2009	Human	Human	SI_1105	Leiden-Kuijper	3.906433
ERS138024	Europe	Spain	Barcelona	2009	Human	Human	ES_1203	Leiden-Kuijper	3.907144
ERS138025	Europe	Switzerland	Basel	2009	Human	Human	CH_0104	Leiden-Kuijper	3.970371
ERS138026	Europe	Netherlands	Leiden	2006	Human	Human	6072310	Leiden-Kuijper	3.89745
ERS138027	Europe	Netherlands	Nijmegen	2006	Human	Human	6086336	Leiden-Kuijper	4.038505
ERS138028	Europe	Netherlands	Leiden	2007	Human	Human	7001233	Leiden-Kuijper	4.072708
ERS138029	Europe	Netherlands	Groningen	2007	Human	Human	7004578	Leiden-Kuijper	4.023331
ERS138030	Europe	Netherlands	Utrecht	2007	Human	Human	7005405	Leiden-Kuijper	3.871749
ERS138031	Europe	Netherlands	Zwolle	2007	Human	Human	7021455	Leiden-Kuijper	3.992912
ERS138032	Europe	Netherlands	Zwolle	2007	Human	Human	7044912	Leiden-Kuijper	3.876673
ERS138033	Europe	Netherlands	Zwolle	2007	Human	Human	7066827	Leiden-Kuijper	4.039112

ERS138034	Europe	Netherlands	Zwolle	2007	Human	Human	7071308	Leiden-Kuijper	4.064735
ERS138035	Europe	Netherlands	Zwolle	2007	Human	Human	7086074	Leiden-Kuijper	3.996044
ERS138037	Europe	Netherlands	Leiden	2008	Human	Human	8011061	Leiden-Kuijper	4.011475
ERS138038	Europe	Netherlands	Utrecht	2008	Human	Human	8013820	Leiden-Kuijper	3.959776
ERS138039	Europe	Netherlands	Leiden	2008	Human	Human	8051728	Leiden-Kuijper	3.835557
ERS138040	Europe	Netherlands	Leiden	2008	Human	Human	8055344	Leiden-Kuijper	3.938877
ERS138042	Europe	Netherlands	Breda	2008	Human	Human	8091554	Leiden-Kuijper	3.93788
ERS138043	Europe	Netherlands	Harderwijk	2009	Human	Human	9012668	Leiden-Kuijper	3.833424
ERS138044	Europe	Netherlands	Goes	2009	Human	Human	9019497	Leiden-Kuijper	3.833188
ERS138045	Europe	Netherlands	Hoorn	2009	Human	Human	9077637	Leiden-Kuijper	4.083592
ERS138046	Europe	Netherlands	Roermond	2010	Human	Human	10005075	Leiden-Kuijper	4.079046
ERS138047	Europe	Netherlands	Rotterdam	2010	Human	Human	10015222	Leiden-Kuijper	4.009925
ERS138048	Europe	Netherlands	Velp	2010	Human	Human	10080193	Leiden-Kuijper	4.065455
ERS138049	Europe	Netherlands	Zeeland	2011	Human	Human	11012929	Leiden-Kuijper	4.09807
ERS138051	Europe	Netherlands	Zwolle	2009	Human	Human	NL_1103	Leiden-Kuijper	4.082329
ERS138052	Europe	Netherlands	Gastel	2009	Animal	Swine	P29	Leiden-Kuijper	4.120959
ERS138053	Europe	Netherlands	NI	2009	Animal	Swine	P60	Leiden-Kuijper	3.964137
ERS138054	Europe	Netherlands	Flevoland	2009	Animal	Swine	P27	Leiden-Kuijper	3.947963
ERS138055	Europe	Netherlands	NI	2009	Animal	Swine	P70	Leiden-Kuijper	4.050756
ERS138056	Europe	Netherlands	Tolakker	2009	Animal	Swine	P52	Leiden-Kuijper	3.936637
ERS138057	Europe	UK	Glasgow	2009	Human	Human	UK_1302	Leiden-Kuijper	3.939525
ERS138063	Europe	Netherlands	Netherlands	2002	Human	Human	126065	Leiden-Kuijper	3.990764
ERS138064	Europe	Netherlands	Netherlands	2002	Human	Human	126819	Leiden-Kuijper	3.941533
ERS138065	Europe	Netherlands	Netherlands	2002	Human	Human	126938	Leiden-Kuijper	3.940558
ERS138066	Europe	Netherlands	Netherlands	2002	Human	Human	129820	Leiden-Kuijper	3.865841

ERS138067	Europe	Netherlands	Netherlands	2010	Animal	horse	cvi52	Leiden-Kuijper	4.082987
ERS138068	Europe	Netherlands	Netherlands	2010	Animal	horse	cvi53	Leiden-Kuijper	4.08574
ERS138069	Europe	Netherlands	Aarle-Rixtel	2011	human	human	H205	Leiden-Kuijper	3.869255
ERS138070	Europe	Netherlands	Aarle-Rixtel	2011	Animal	Swine	B37.2	Leiden-Kuijper	3.866941
ERS138073	Europe	Netherlands	Netherlands	2010	Human	Human	53737	Leiden-Kuijper	3.950177
ERS138074	Europe	Netherlands	Netherlands	2007	Human	Human	47337	Leiden-Kuijper	3.894323
ERS138087	N-America	Ontario_Hospital_MS	NI	2005	Human	Human	MOH649	Guelph-Weese	4.042571
ERS138088	N-America	Ontario_Hospital-k	NI	2005	Human	Human	MOH673	Guelph-Weese	3.876956
ERS138089	N-America	Ontario_Hospital-HDGH	NI	2005	Human	Human	MOH887	Guelph-Weese	4.002402
ERS138090	N-America	Ontario_Hospital-MS	NI	2005	Human	Human	M0H744	Guelph-Weese	4.047394
ERS138092	Asia	Iran	NI	2011	Human	Human	MJ20	Guelph-Weese	3.941343
ERS138093	Asia	Iran	NI	2011	Human	Human	MJ38	Guelph-Weese	4.042855
ERS138094	Asia	Iran	NI	2011	Human	Human	MJ39	Guelph-Weese	3.981697
ERS138095	N-America	Ontario_Hospital_MS	NI	2004	Human	Human	Cd247	Guelph-Weese	3.83856
ERS138096	N-America	Ontario_Hospital_MS	NI	2004	Human	Human	Cd357	Guelph-Weese	3.9295
ERS138097	N-America	Ontario_Hospital_JB	NI	2008	Human	Human	Cd901	Guelph-Weese	3.914076
ERS138098	N-America	Ontario_Hospital_B	NI	2008	Human	Human	Cd973	Guelph-Weese	3.873811
ERS138099	N-America	Ontario_Hospital_K	NI	2011	Human	Human	MF958	Guelph-Weese	3.918145
ERS138100	N-America	Ontario_Hospital_CH	NI	2011	Human	Human	CHP20	Guelph-Weese	3.841065
ERS138103	N-America	Manitoba	NI	2008	Animal	Swine	Cd1082	Guelph-Weese	3.963425
ERS138104	N-America	Ontario	NI	2008	Animal	Swine	Cd1085	Guelph-Weese	3.963828
ERS138105	N-America	Saskatchewan	NI	2009	Animal	Swine	Cd1087	Guelph-Weese	3.967526
ERS138107	N-America	Ontario	NI	2009	Animal	Swine	Cd1095	Guelph-Weese	3.964246
ERS138115	N-America	Case-control_study_2004	NI	2004	Animal	Bovine	Cd729	Guelph-Weese	4.002431
ERS138116	N-America	Case-control_study_2004	NI	2004	Animal	Bovine	Cd732	Guelph-Weese	3.980178

ERS138117	N-America	Case-control_study_2004	NI	2004	Animal	Bovine	Cd739	Guelph-Weese	3.846505
ERS138118	N-America	Case-control_study_2004	NI	2004	Animal	Bovine	Cd745	Guelph-Weese	3.908312
ERS138119	N-America	Case-control_study_2004	NI	2004	Animal	Bovine	Cd757	Guelph-Weese	3.920078
ERS138120	N-America	Ontario	NI	2010	Animal	Equine	AS-le	Guelph-Weese	3.845395
ERS138122	N-America	Ontario	NI	2010	Animal	Equine	AS-VI-2	Guelph-Weese	3.905424
ERS138124	N-America	Ontario	NI	2008	Food	Chicken_thigh	Cd1014	Guelph-Weese	3.840168
ERS138125	N-America	Ontario	NI	2008	Food	Chicken_leg	Cd1035	Guelph-Weese	3.956672
ERS138126	N-America	Ontario	NI	2008	Food	Chicken_wing	Cd1037	Guelph-Weese	3.957376
ERS138127	N-America	Ontario	NI	2008	Food	Chicken_thigh	Cd1069	Guelph-Weese	3.834714
ERS138130	Europe	UK	Liverpool	2009	Human	Human	CDP080037	Liverpool-F. Miyajima	3.95866
ERS138132	Europe	UK	Liverpool	2009	Human	Human	CDN080535	Liverpool-F. Miyajima	3.961183
ERS138133	Europe	UK	Liverpool	2009	Human	Human	43236_X	Liverpool-F. Miyajima	3.911063
ERS138134	Europe	UK	Liverpool	2009	Human	Human	25047_J	Liverpool-F. Miyajima	4.029163
ERS138135	Europe	UK	Liverpool	2009	Human	Human	CDP080060	Liverpool-F. Miyajima	4.086043
ERS138136	Europe	UK	Liverpool	2009	Human	Human	CDN080571	Liverpool-F. Miyajima	3.978151
ERS138137	Europe	UK	Liverpool	2009	Human	Human	CDN080575	Liverpool-F. Miyajima	3.956806
ERS138138	Europe	UK	Liverpool	2010	Human	Human	CDN080576	Liverpool-F. Miyajima	3.871238
ERS138139	Europe	UK	Liverpool	2010	Human	Human	CDN080581	Liverpool-F. Miyajima	3.9568
ERS138140	Europe	UK	Liverpool	2010	Human	Human	CDN080584	Liverpool-F. Miyajima	3.994223
ERS138141	Europe	UK	Liverpool	2010	Human	Human	CDN080585	Liverpool-F. Miyajima	3.8681
ERS138142	Europe	UK	Liverpool	2010	Human	Human	CDP080084	Liverpool-F. Miyajima	3.87666
ERS138143	Europe	UK	Liverpool	2010	Human	Human	CDN080590	Liverpool-F. Miyajima	3.961571
ERS138144	Europe	UK	Liverpool	2010	Human	Human	CDP080088	Liverpool-F. Miyajima	3.851189
ERS138145	Europe	UK	Liverpool	2010	Human	Human	CDP080097	Liverpool-F. Miyajima	3.90988
ERS138146	Europe	UK	Liverpool	2010	Human	Human	CDN080599	Liverpool-F. Miyajima	3.907774

ERS138147	Europe	UK	Liverpool	2010	Human	Human	CDP080102	Liverpool-F. Miyajima	3.963185
ERS138148	Europe	UK	Liverpool	2010	Human	Human	CDP080108	Liverpool-F. Miyajima	3.958722
ERS138149	Europe	UK	Liverpool	2010	Human	Human	CDP080109	Liverpool-F. Miyajima	3.889673
ERS138151	Europe	UK	Liverpool	2011	Human	Human	13928.H	Liverpool-F. Miyajima	3.938534
ERS138152	Europe	UK	Liverpool	2011	Human	Human	16182.P	Liverpool-F. Miyajima	3.960883
ERS138154	Europe	UK	Liverpool	2011	Human	Human	52357.D	Liverpool-F. Miyajima	4.031947
ERS1645161	Europe	UK	Cardiff	1996	Human	Human		Cardiff- T. Connor	3.975253
ERS1645162	Europe	UK	Cardiff	1996	Human	Human		Cardiff- T. Connor	4.100392
ERS1645163	Europe	UK	Cardiff	1997	Human	Human		Cardiff- T. Connor	3.966632
ERS1645164	Europe	UK	Cardiff	1997	Human	Human		Cardiff- T. Connor	4.105619
ERS1645165	Europe	UK	Cardiff	1997	Human	Human		Cardiff- T. Connor	3.966245
ERS1645166	Europe	UK	Cardiff	1997	Human	Human		Cardiff- T. Connor	3.930095
ERS1645167	Europe	UK	Cardiff	1998	Human	Human		Cardiff- T. Connor	4.019092
ERS1645168	Europe	UK	Cardiff	1994	Human	Human		Cardiff- T. Connor	3.928639
ERS188478	N-America	Iowa	NI	1999	Animal	Swine	382	Iowa-Songer	3.891978
ERS188479	N-America	Iowa	NI	2000	Animal	Swine	449	Iowa-Songer	3.907055
ERS188480	N-America	Iowa	NI	2001	Animal	Swine	645	Iowa-Songer	3.851929
ERS188481	N-America	Iowa	NI	2001	Animal	Swine	649	Iowa-Songer	3.899151
ERS188482	N-America	Iowa	NI	2002	Animal	Swine	652	Iowa-Songer	3.945244
ERS188483	N-America	Iowa	NI	2002	Animal	Swine	685	Iowa-Songer	3.988367
ERS188484	N-America	Iowa	NI	2002	Animal	Swine	696	Iowa-Songer	3.928586
ERS188485	N-America	Iowa	NI	2006	Animal	Bovine	697	Iowa-Songer	3.968063
ERS188486	N-America	Iowa	NI	2006	Animal	Bovine	701	Iowa-Songer	3.893246
ERS188487	N-America	Iowa	NI	2006	Animal	Bovine	706	Iowa-Songer	3.993404
ERS188489	N-America	Iowa	NI	2002	Animal	Swine	734	Iowa-Songer	3.942151

ERS188492	N-America	Iowa	NI	2002	Animal	Swine	778	Iowa-Songer	3.846361
ERS188493	N-America	Iowa	NI	2002	Animal	Bovine	796	Iowa-Songer	3.975672
ERS188494	N-America	Iowa	NI	2002	Animal	Swine	801	Iowa-Songer	3.993712
ERS188495	N-America	Iowa	NI	2002	Animal	Bovine	817	Iowa-Songer	3.954906
ERS188498	N-America	Iowa	NI	2002	Animal	Bovine	854	Iowa-Songer	3.969691
ERS188502	N-America	Iowa	NI	2006	Human	Human	6180	Iowa-Songer	3.991632
ERS188503	N-America	Iowa	NI	2006	Human	Human	6181	Iowa-Songer	3.851683
ERS188504	N-America	Iowa	NI	2006	Human	Human	6182	Iowa-Songer	3.850761
ERS188534	Australia	Australia	Sydney	2011	Human	Human	ES_471	Monash-Riley	3.961765
ERS188555	Europe	UK	Dudley	2007	Human	Human	078.1	Birmingham	3.950124
ERS188556	Europe	UK	Staffordshire	2007	Human	Human	078.2	Birmingham	3.891632
ERS188557	Europe	UK	Dudley	2008	Human	Human	078.3	Birmingham	3.933567
ERS188558	Europe	UK	Birmingham	2008	Human	Human	078.4	Birmingham	3.885592
ERS188559	Europe	UK	Coventry	2008	Human	Human	078.5	Birmingham	3.937331
ERS188560	Europe	UK	Lincolnshire	2008	Human	Human	078.6	Birmingham	3.821256
ERS188561	Europe	UK	Shrewsbury	2008	Human	Human	078.7	Birmingham	4.00415
ERS188562	Europe	UK	Nottinghamshire	2008	Human	Human	078.8	Birmingham	3.825363
ERS188563	Europe	UK	Derbyshire	2009	Human	Human	078.9	Birmingham	3.935596
ERS188564	Europe	UK	Worcestershire	2009	Human	Human	078.10	Birmingham	3.887548
ERS188565	Europe	UK	Walsall	2009	Human	Human	078.11	Birmingham	3.8495
ERS188566	Europe	UK	Nottinghamshire	2009	Human	Human	078.12	Birmingham	3.931376
ERS188567	Europe	UK	Worcestershire	2010	Human	Human	078.13	Birmingham	3.823766
ERS188569	Europe	UK	Shrewsbury	2010	Human	Human	078.15	Birmingham	3.889828
ERS188570	Europe	UK	Shrewsbury	2010	Human	Human	078.16	Birmingham	3.941903
ERS188571	Europe	UK	Shrewsbury	2010	Human	Human	078.17	Birmingham	3.93559

ERS188572	Europe	UK	Shrewsbury	2010	Human	Human	078.18	Birmingham	3.840718
ERS188573	Europe	UK	Northants	2010	Human	Human	078.19	Birmingham	3.916189
ERS188574	Europe	UK	Nottinghamshire	2010	Human	Human	078.20	Birmingham	3.896075
ERS188575	Europe	UK	Northants	2010	Human	Human	078.21	Birmingham	4.036098
ERS188576	Europe	UK	Northants	2011	Human	Human	078.22	Birmingham	3.866922
ERS188577	Europe	UK	Shrewsbury	2011	Human	Human	078.23	Birmingham	3.840113
ERS188578	Europe	UK	Shrewsbury	2011	Human	Human	078.24	Birmingham	3.962682
ERS188579	Europe	UK	Shrewsbury	2011	Human	Human	078.25	Birmingham	3.962219
ERS188580	Europe	UK	Staffordshire	2011	Human	Human	078.26	Birmingham	4.008034
ERS188581	Europe	UK	Northants	2011	Human	Human	078.27	Birmingham	3.870671
ERS188582	Europe	UK	Walsall	2011	Human	Human	078.28	Birmingham	3.848588
ERS188583	Europe	UK	Shrewsbury	2011	Human	Human	078.29	Birmingham	3.963406
ERS188584	Europe	UK	Lincolnshire	2011	Human	Human	078.30	Birmingham	3.962778
ERS188585	Europe	UK	Birmingham	2011	Human	Human	078.31	Birmingham	3.919167
ERS188586	Europe	UK	Shrewsbury	2009	Human	Human	078.32	Birmingham	3.964696
ERS188587	Europe	UK	Dudley	2011	Human	Human	078.33	Birmingham	3.903676
ERS188588	Europe	UK	Dudley	2011	Human	Human	078.34	Birmingham	3.97005
ERS188589	Europe	UK	Derbyshire	2011	Human	Human	078.35	Birmingham	3.907765
ERS188590	Europe	UK	Nottinghamshire	2011	Human	Human	078.36	Birmingham	3.835469
ERS188591	Europe	UK	Shrewsbury	2012	Human	Human	078.37	Birmingham	3.958636
ERS188592	Europe	UK	Coventry	2012	Human	Human	078.38	Birmingham	4.049715
ERS188658	Europe	Glasgow	EDI-CML	2008	Human	Human	20080322	Glasgow	3.94181
ERS188659	Europe	Glasgow	GLA-ROY	2008	Human	Human	20080936	Glasgow	3.961665
ERS188660	Europe	Glasgow	ABD-ROY	2010	Human	Human	20100862	Glasgow	4.021448
ERS188661	Europe	Glasgow	EDI-ROY	2012	Human	Human	20120077	Glasgow	3.869846

ERS188662	Europe	Glasgow	EDI-ROY	2012	Human	Human	20120080	Glasgow	3.867824
ERS188663	Europe	Glasgow	GLA-ROY	2012	Human	Human	20120423	Glasgow	4.11007
ERS188664	Europe	Glasgow	INV-RAI	2012	Human	Human	20120540	Glasgow	3.963186
ERS188665	Europe	Glasgow	EDI-ROY	2012	Human	Human	20120569	Glasgow	3.864738
ERS188666	Europe	Glasgow	EDI-ROY	2012	Human	Human	20120812	Glasgow	3.867326
ERS188667	Europe	Glasgow	AYR-ARL	2012	Human	Human	20120873	Glasgow	3.866129
ERS199743	Europe	Austria	NI	2010	Animal	Calf	910150/2010	Austria-Indra	3.901198
ERS199744	Europe	Austria	NI	2012	Animal	Swine	910500/2012	Austria-Indra	4.041694
ERS199746	Europe	Austria	NI	2012	Animal	Swine	910503/2012	Austria-Indra	4.039241
ERS199747	Europe	Austria	Vienna	2012	human	human	910511/2012	Austria-Indra	4.085182
ERS199748	Europe	Austria	Tyrol	2012	human	human	910528/2012	Austria-Indra	4.015643
ERS199749	Europe	Austria	Styria	2012	human	human	910551/2012	Austria-Indra	4.008419
ERS199750	Europe	Belgium	NI	2010	Animal	Calf	S4	Slovenia- M. Rupnik	4.054965
ERS199751	Europe	Belgium	NI	2010	Animal	Calf	S8	Slovenia- M. Rupnik	4.05101
ERS199753	Europe	Belgium	NI	2010	Animal	Calf	S12	Slovenia- M. Rupnik	3.88543
ERS199755	Europe	Belgium	NI	2010	Animal	Calf	S16	Slovenia- M. Rupnik	3.882253
ERS199756	Europe	Belgium	NI	2010	Animal	Calf	S17	Slovenia- M. Rupnik	4.057932
ERS199759	Europe	Belgium	NI	2010	Animal	Calf	S23	Slovenia- M. Rupnik	4.052452
ERS199762	Europe	Belgium	NI	2010	Animal	Calf	S27	Slovenia- M. Rupnik	4.121371
ERS199765	Europe	Slovenia	NI	2009	Environment	River_water	ZZV09-2266	Slovenia- M. Rupnik	3.924512
ERS199786	Europe	Netherlands	Lemele	2011	Human	farmer	H102	Leiden-Kuijper	3.97726
ERS199787	Europe	Netherlands	Raamsdonkveer	2011	Animal	Swine	B31.3	Leiden-Kuijper	4.034344
ERS199788	Europe	Netherlands	Heino	2011	Animal	Swine	B17.3	Leiden-Kuijper	3.947923
ERS199789	Europe	Netherlands	Ulft	2011	Human	farmer	H121	Leiden-Kuijper	3.965174
ERS199790	Europe	Netherlands	Rijen	2011	Animal	Swine	B27.7	Leiden-Kuijper	4.067124

ERS199791	Europe	Netherlands	Baarle-Nassau	2011	Human	farmer	H230	Leiden-Kuijper	4.046339
ERS199792	Europe	Netherlands	Oirschot	2011	Human	farmer	H189	Leiden-Kuijper	4.068827
ERS199793	Europe	Netherlands	Lierop	2011	Animal	Swine	B23.6	Leiden-Kuijper	4.088705
ERS199794	Europe	Netherlands	Aarle-Rixtel	2011	Human	farmer	H205	Leiden-Kuijper	3.885643
ERS199795	Europe	Netherlands	Hardenberg	2011	Animal	Swine	B15.1	Leiden-Kuijper	3.992438
ERS199796	Europe	Netherlands	NI	2011	Human	farmer	H21	Leiden-Kuijper	4.021918
ERS199797	Europe	Netherlands	Oirschot	2011	Animal	Swine	B30.5	Leiden-Kuijper	3.950647
ERS199798	Europe	Netherlands	Rijen	2011	Human	farmer	H122	Leiden-Kuijper	3.876074
ERS199799	Europe	Netherlands	Hardenberg	2011	Human	farmer	H95	Leiden-Kuijper	3.982881
ERS199800	Europe	Netherlands	Raamsdonk	2011	Animal	Swine	B28.1	Leiden-Kuijper	4.134619
ERS199801	Europe	Netherlands	NI	2011	Human	farmer	H214	Leiden-Kuijper	3.87767
ERS199802	Europe	Netherlands	NI	2011	Human	farmer	H258	Leiden-Kuijper	4.032468
ERS199803	Europe	Netherlands	Heino	2011	Human	farmer	H88	Leiden-Kuijper	3.949852
ERS199804	Europe	Netherlands	NI	2011	Human	farmer	H111	Leiden-Kuijper	4.002207
ERS199805	Europe	Netherlands	Baarle-Nassau	2011	Animal	Swine	B39.4	Leiden-Kuijper	4.056245
ERS199806	Europe	Netherlands	Moergestel	2011	Animal	Swine	B4.2	Leiden-Kuijper	3.907108
ERS199807	Europe	Netherlands	Lemele	2011	Animal	Swine	B20.1	Leiden-Kuijper	3.948195
ERS199809	Europe	Netherlands	Aarle-Rixtel	2011	Animal	Swine	B37.3	Leiden-Kuijper	3.886593
ERS199810	Europe	Netherlands	Ermelo	2011	Animal	Swine	B1.5	Leiden-Kuijper	3.989958
ERS199811	Europe	Netherlands	Lierop	2011	Human	farmer	H170	Leiden-Kuijper	3.962313
ERS199812	Europe	Netherlands	Ulft	2011	Animal	Swine	B22.6	Leiden-Kuijper	4.04709
ERS199813	Europe	Netherlands	Houten	2011	Animal	Swine	B29.10	Leiden-Kuijper	3.837711
ERS199814	Europe	Netherlands	Houten	2011	Human	farmer	H141	Leiden-Kuijper	3.83805
ERS199826	Europe	Italy	Torino	2009	Human	Human	IT_0109	Leiden-Kuijper	4.069792
ERS199827	Europe	UK	Leeds	2008	Human	Human	57/33	Leiden-Kuijper	3.898584

ERS362924	Europe	Netherlands	Arnhem	2008	Human	Human	8056692	Leiden-Kuijper	4.072006
